# Principal component gene set enrichment (PCGSE)

**DOI:** 10.1186/s13040-015-0059-z

**Published:** 2015-08-19

**Authors:** H. Robert Frost, Zhigang Li, Jason H. Moore

**Affiliations:** 1 Institute of Quantitative Biomedical Sciences, Geisel School of Medicine, Lebanon, 03756 NH USA; 2 Section of Biostatistics and Epidemiology, Department of Community and Family Medicine, Geisel School of Medicine, Lebanon, 03756 NH USA; 3 Department of Genetics, Dartmouth College, Hanover, 03755 NH USA

## Abstract

**Background:**

Although principal component analysis (PCA) is widely used for the dimensional reduction of biomedical data, interpretation of PCA results remains daunting. Most existing interpretation methods attempt to explain each principal component (PC) in terms of a small number of variables by generating approximate PCs with mainly zero loadings. Although useful when just a few variables dominate the population PCs, these methods can perform poorly on genomic data, where interesting biological features are frequently represented by the combined signal of functionally related sets of genes. While gene set testing methods have been widely used in supervised settings to quantify the association of groups of genes with clinical outcomes, these methods have seen only limited application for testing the enrichment of gene sets relative to sample PCs.

**Results:**

We describe a novel approach, principal component gene set enrichment (PCGSE), for unsupervised gene set testing relative to the sample PCs of genomic data. The PCGSE method computes the statistical association between gene sets and individual PCs using a two-stage competitive gene set test. To demonstrate the efficacy of the PCGSE method, we use simulated and real gene expression data to evaluate the performance of various gene set test statistics and significance tests.

**Conclusions:**

Gene set testing is an effective approach for interpreting the PCs of high-dimensional genomic data. As shown using both simulated and real datasets, the PCGSE method can generate biologically meaningful and computationally efficient results via a two-stage, competitive parametric test that correctly accounts for inter-gene correlation.

**Electronic supplementary material:**

The online version of this article (doi:10.1186/s13040-015-0059-z) contains supplementary material, which is available to authorized users.

## Background

PCA is a well established statistical technique that performs a linear transformation of multivariate data into a new set of variables, the principal components (PCs), that are linear combinations of the original variables, are uncorrelated and have sequentially maximum variance [1–3]. The solution to PCA is given by the spectral decomposition of the covariance matrix with the variance of the PCs specified by the eigenvalues, arranged in decreasing order, and the PC directions specified by the associated eigenvectors.

In the biomedical domain, PCA has been extensively employed for the analysis of genomic data including measures of DNA variation, DNA methylation and RNA expression [4]. Features of these datasets that motivate PCA include the high dimensionality of the feature space, low sample size and significant collinearity. The most common uses of PCA with genomic data involve dimensionality reduction for visualization [5, 6] or clustering of the observations [7], with population genetics an important use case [8]. PCA has also been used as the basis for feature selection [9], gene clustering [10] and bi-clustering [11]. More recent applications include dimensionality reduction prior to gene set testing [12, 13] and high-dimensional regression [14].

Although PCA is an effective tool for reducing the dimensionality of genomic data, application of the method remains limited by the challenge of biological interpretation [4, 15]. Because PCs are linear combinations of all original variables, which can number from the thousands to the millions for genomic datasets, they typically lack any clear biological meaning. While PCA may improve the performance of many statistical methods, e.g., better predictive accuracy in a regression context, the underlying model is often a black box.

Approaches for generating more interpretable PCs have evolved from component thresholding [3], simple components (i.e., PC loading vectors constrained to values from {−1,0,1}) [16] and rotation techniques (e.g., varimax) [17] to sparse PCA methods, which compute approximate PCs using cardinality [18] or LASSO-based [15, 19] constraints on the component loadings. By generating approximate PCs with few non-zero loadings, all of these techniques improve interpretability by associating only a small number of variables with each PC. While such sparse PCA methods can be very effective when the true population PCs are associated with only a few variables, they will fail to accurately estimate the spectral structure of the data when the population PCs are defined by the coordinated action of large groups of variables with small marginal effects. For genomic data, the pathway-based patterns that dominate the robust structure of genetic associations with clinical phenotypes [20], and are the motivation for traditional gene set testing methods [21], can be expected to also characterize the PCs of those datasets. The PCs of genomic data are therefore more likely to be quantitatively described, in a repeatable fashion, by collections of functionally related genes, e.g., gene sets from the Gene Ontology (GO) [22], than by individual genes.

To support interpretation of PCs in terms of *a priori* variable groups, rather than just individual variables, sparse PCA methods have recently been extended to include structured sparse penalties [23, 24], such as the group lasso [25]. Although structured sparse PCA techniques generate sparse PC loading vectors that reflect group structure, these methods cannot be easily used to compute the statistical association between variable groups and each PC in such a way that the variable groups can be ranked according to deviation from a specific null hypothesis, as is done in traditional gene set testing. Matrix correlation methods [3, 26] have also been used to quantify the association between groups of variables and one or more PCs. However, because such matrix correlation methods compute the association of each variable group independent of the variables that do not belong to the group, they can only be used for self-contained gene set tests [27] (*Q*_2_ in the terminology of Tian et al. [28]) in a manner similar to Goeman and Buhmann’s *globaltest* [29] and not for competitive gene set testing (*Q*_1_ in the terminology of Tian et al.).

To date, competitive gene set testing relative to PCs has been limited to methods, such as Fisher’s Exact Test, that are based on a 2×2 contingency table representing the association between gene set membership and a discretization of the ranked list of PC loading values [30]. Such contingency table tests have two key flaws: they rely on an arbitrary threshold of the gene-level test statistic, which reduces statistical power and, more importantly, they are based on the incorrect assumption of independence among the gene-level test statistics, causing them to generate high type I error rates [27, 31, 32]. Because of the anti-conservative nature of contingency table-based tests, and other approaches that assume independence among gene-level test statistics under the null, the use of these methods for standard gene set testing has been strongly discouraged in favor of techniques that preserve inter-gene correlation, usually via permutation of the sample labels [27]. Competitive gene set testing methods that correctly account for correlation among gene-level test statistics, either through sample permutation, parametric approximation of the sample permutation distribution or correlation adjustment of parametric test statistics, include SAFE [31, 33, 34], GSEA [35], GSA [36] and CAMERA [32]. All of these methods, however, are designed for use in a supervised context to measure the statistical significance of the association between sets of genomic variables and a phenotype variable.

Although biologically meaningful and repeatable interpretation of the PCs of genomic data requires approaches based on functional gene sets, researchers currently lack methods that competitively test the association between gene sets and PCs with correct handling of inter-gene correlation. To address this gap, we have developed principal component gene set enrichment (PCGSE), an approach for interpreting the PCs of genomic data via two-stage competitive gene set testing in which the correlation between each gene and each PC is used as a gene-level statistic with flexible choice of both the gene set test statistic and the method used to compute the null distribution of the gene set statistic. Although described in the context of functional gene sets and genomic data, the PCGSE method can be used to compute the statistical association between any collection of variable groups and the PCs of an empirical dataset. To support use of the PCGSE method by other researchers, we implemented the *PCGSE* R package, which is available from the CRAN repository. Using simulated data with simulated gene sets and real gene expression data with curated gene sets, we demonstrate that biologically meaningful and computationally efficient results can be obtained from a simple parametric version of the PCGSE technique, based on the CAMERA method [32], that performs a correlation-adjusted two-sample t-test between the gene-level test statistics for gene set members and genes not in the set.

## Methods

### PCGSE inputs

The PCGSE method takes as input both an *n*×*p* genomic data matrix **X** quantifying *p* genomic variables under *n* experimental conditions and an *f*×*p* binary annotation matrix **A** that specifies the association between the *p* genomic variables and *f* functional categories.

The genomic data held in **X**, e.g., mRNA expression levels, will be modeled as a sample of *n* independent observations from a *p*-dimensional random vector **x**. Although PCGSE does not have specific distributional requirements, sources of genomic data, especially gene expression data, are often well approximated by a multivariate normal distribution after appropriate transformations. It is assumed that any desired data transformations have been performed and that missing values have been imputed or removed.

The rows of the annotation matrix **A** represent *f* distinct biological functions, e.g., GO categories, and the elements *a*_*i*,*j*_ hold indicator variables whose value depends on whether an annotation exists between the function *i* and genomic variable *j*.

### PCGSE algorithm

Enrichment of the gene sets defined by **A** relative to one of the PCs of **X** is performed using the following sequence of steps. This workflow is graphically illustrated in Fig. 1 and each step is explained in more detail in the Sections “PCA for PCGSE” thru “Gene set statistical significance” below. Note that steps 2 thru 5 have close parallels to modules in Ackermann and Strimmer’s general modular framework for gene set enrichment analysis [37].

1. Perform PCA on a standardized version of **X**.

**Fig. 1:**
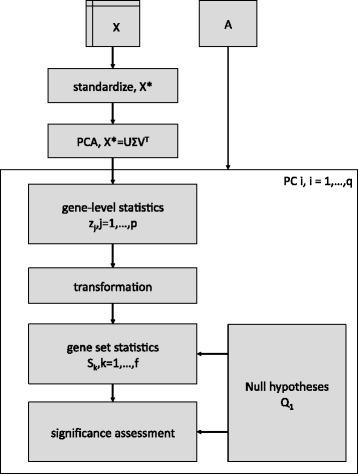
PCGSE algorithm. Illustration of the PCGSE algorithm as outlined in Section “PCGSE algorithm”. This schematic is based on the general gene set testing workflow of Ackermann and Strimmer [37]
2. Compute gene-level statistics, *z*_*j*_,*j*=1,…,*p*, for all *p* genomic variables that quantify the association between the genomic variable and the PC.
3. (Optional) Transform the gene-level statistics.
4. Compute gene set statistics, *S*_*k*_,*k*=1,…,*f*, for all *f* gene sets defined by **A** using the gene-level statistics, *z*_*j*_.
5. Determine the statistical significance of the gene set statistics according to a competitive null hypothesis.

*Output:* For each of the *f* gene sets, the observed value of the gene set test statistic, *S*_*k*_, and a p-value representing the probability of encountering a gene set statistic as or more extreme than then the observed *S*_*k*_ under the appropriate competitive null hypothesis.

### PCA for PCGSE

Because PCs are not invariant under scaling of the data [3], PCA is performed on a mean centered and standardized version of **X**, $\tilde {\mathbf {X}}$. The PC loading vectors and variances of $\tilde {\mathbf {X}}$ are thus the eigenvectors and eigenvalues of the sample correlation matrix, $\mathbf {S} = 1/(n-1) \tilde {\mathbf {X}}^{T} \tilde {\mathbf {X}}$, rather than the sample covariance matrix. For computational efficiency, the PCA solution is realized via the singular value decomposition (SVD) of $\tilde {\mathbf {X}}$, $\tilde {\mathbf {X}} = \mathbf {U} \boldsymbol {\Sigma } \mathbf {V}^{T}$, where the columns of **V** represent the PC loading vectors, the entries in the diagonal matrix ***Σ*** are proportional to the square roots of the PC variances and the columns of **U*Σ*** are the PCs.

### Gene-level statistics

The PCGSE method supports the following gene-level statistics, represented using the notation *z*_*j*_,*j*=1,…,*p*, for quantifying the association between genomic variable *j* and the target PC.

- *PC loading.* For genomic variable *j* and target PC *m*, the gene-level statistic is element *v*_*j*,*m*_ of matrix **V** from the SVD of $\tilde {\mathbf {X}}$.
- *Pearson correlation coefficient.* Where the correlation is computed between each genomic variable and the target PC.
- *Fisher-transformed Pearson correlation coefficient.* This creates a statistic whose distribution is approximately $\mathcal {N}(0,1)$.

Because the Pearson correlation coefficients between genomic variables and PCs of the sample correlation matrix are proportional to the PC loadings (see (1) below), all of these gene-level statistics provide a measure of the correlation between genomic variables and PCs. Specifically,

(1) $$ \begin{aligned} \mathbf{P}_{\tilde{\mathbf{X}}, \mathbf{U} \boldsymbol{\Sigma}} &= \mathbf{S}_{\tilde{\mathbf{X}}, \mathbf{U} \boldsymbol{\Sigma} \sqrt{1-n} \boldsymbol{\Sigma}^{-1}} = \frac{1}{n-1} (\mathbf{U} \boldsymbol{\Sigma} \mathbf{V}^{T})^{T} \mathbf{U} \sqrt{1-n} = \frac{1}{\sqrt{n-1}} \mathbf{V} \boldsymbol{\Sigma} \end{aligned}  $$

where **U**,***Σ*** and **V** are from the SVD of $\tilde {\mathbf {X}}$, $\mathbf {P}_{\tilde {\mathbf {X}}, \mathbf {U} \boldsymbol {\Sigma }}$ is the matrix of Pearson correlation coefficients between the standardized genomic variables held in $\tilde {\mathbf {X}}$ and the PCs of $\tilde {\mathbf {X}}$, with element *i*,*j* of $\mathbf {P}_{\tilde {\mathbf {X}}, \mathbf {U} \boldsymbol {\Sigma }}$ set to the Pearson correlation coefficient between column *i* of $\tilde {\mathbf {X}}$ and column *j* of **U*Σ***, and $\mathbf {S}_{\tilde {\mathbf {X}}, \mathbf {U} \boldsymbol {\Sigma } \sqrt {1-n} \boldsymbol {\Sigma }^{-1}}$ is the matrix of sample covariances between the columns of $\tilde {\mathbf {X}}$ and the columns of $\mathbf {U} \boldsymbol {\Sigma } \sqrt {1-n} \boldsymbol {\Sigma }^{-1}$.

The choice between the different gene-level statistics will be guided by the gene set statistic and significance testing method employed for PCGSE as well as computational constraints. For example, the added computational expense to generate z-statistics from correlation coefficients is motivated by parametric tests of the mean difference statistic, whereas, for rank sum tests, the PC loadings are sufficient.

### Transformation of gene-level statistics

An absolute value transformation can optionally be applied to the gene-level statistics, i.e., $\tilde {z}_{j} = |z_{j}|$. Such a transformation gives the PCGSE method increased power to detect scale alternatives, i.e. gene sets that contain both significantly enriched and significantly repressed genomic variables, whereas the use of untransformed gene-level statistics provides better power against shift in location alternatives, i.e., gene sets containing genomic variables with a common direction of association [36].

### Gene set statistics

The PCGSE method supports two competitive gene set statistics, represented using the notation *S*_*k*_,*k*=1,…,*f*, for quantifying the association between gene set *k* and a target PC.

#### Mean difference statistic

This statistic is computed as the standardized difference between the mean of the *z*_*j*_ for genomic variables in the gene set and genomic variables not in the set and corresponds to *U*_*D*_ in the notation of Barry et al. [31]. Benefits of the mean difference statistic include its parametric null distribution and excellent power, relative to other gene set test statistics, for shift in location alternatives when using untransformed *z*_*j*_ [36]. For gene set *k*, this statistic is defined as:

(2) $$ {S^{D}_{k}} = \frac{\bar{z}_{k} - \bar{z}_{k^{c}}}{\sigma_{p} \sqrt{\frac{1}{m_{k}} - \frac{1}{p-m_{k}}}}  $$

where *m*_*k*_ is the number of genes in set *k*, $\bar {z}_{k}$ is the mean of the *z*_*j*_ for members of gene set *k*, $\bar {z}_{k^{c}}$ is the mean of the *z*_*j*_ for genes not in set *k* and *σ*_*p*_ is the pooled standard deviation of the *z*_*j*_.

#### Rank sum statistic

This statistic is computed as the standardized Wilcoxon rank sum statistic given the ranks of the *z*_*j*_ for genomic variables in the set and genomic variables not in the set and corresponds to *U*_*W*_ in the notation of Barry et al. [31]. Benefits of the rank sum statistic include lack of distributional assumptions and robustness to outliers. For gene set *k*, the Wilcoxon rank sum statistic is defined as the sum of the ranks of the gene-level statistic for all genomic variables belonging to gene set *k* minus the minimum possible value for this sum of ranks: $W_{k} = \sum _{j =1}^{p} a_{k,j} \text {Rank}(z_{j}) - \frac {m_{k} (m_{k} +1)}{2}$, where $m_{k} = \sum _{j =1}^{p} a_{k,j}$, the size of gene set *k*. A version of this statistic that has an asymptotic $\mathcal {N}(0,1)$ distribution under the null can be generated as:

(3) $$ {S^{W}_{k}} = \frac{W_{k} - \mu_{W_{k}}}{\sigma^{2}_{W_{k}}}  $$

where $\mu _{W_{k}} = (m_{k}(p-m_{k}))/2$ and $\sigma ^{2}_{W_{k}} = (m_{k}(p-m_{k})(m_{k}+1))/12$.

### Gene set statistical significance

To compute the statistical significance of the association between gene set *k* and a target PC, the distribution of the gene set statistic *S*_*k*_ must be calculated under the appropriate null hypothesis. The PCGSE approach supports three different methods (parametric, correlation-adjusted parametric and permutation) for computing the competitive null distributions of the standardized mean difference statistic, ${S^{D}_{k}}$, and standardized rank sum statistic, ${S^{W}_{k}}$, defined in (2) and (3) respectively.

#### Parametric tests

Under the competitive *H*_0_ that the *z*_*j*_ are i.i.d, ${S^{D}_{k}}$ has a t-distribution with *p*−2 df and a two-sided t-test can therefore be used to determine statistical significance. For ${S^{W}_{k}}$, the asymptotic standard normal distribution under this *H*_0_ can be used as the basis for a two-sided z-test. Both of these parametric tests fall into the class 1 test category as outlined in Barry et al. [31] and are similar to the *Q*_1_ test defined by Tian et al. [28].

While it is often safe to assume a normal distribution for the *z*_*j*_, especially after transformation, the *z*_*j*_ will not be independent. Indeed, because the *z*_*j*_ used with PCGSE are proportional to the PC loadings, they have an asymptotic multivariate normal distribution [38], assuming multivariate normality for the underlying genomic data, with significant correlation present between the loadings associated with the genes that have high pair-wise correlations [3]. Because both the t-test for ${S^{D}_{k}}$ and the z-test for ${S^{W}_{k}}$ ignore this correlation between the *z*_*j*_, they will generate inflated type I error rates. These tests are therefore only supported by the PCGSE method for the purpose of comparative evaluation.

#### Correlation-adjusted parametric tests

A computationally efficient approach for addressing correlation among the *z*_*j*_ involves the use of correlation-adjusted parametric tests. Correlation-adjusted versions of ${S^{D}_{k}}$ and ${S^{W}_{k}}$ were first discussed in the context of gene set testing by Barry et al. [31]. Simplified versions of these correlation-adjusted statistics were later developed into the CAMERA method by Wu et al. [32]. Specifically, the approach taken by CAMERA assumes that correlation among the *z*_*j*_ can be approximated by the correlation among the genomic variables (this is supported by results in Barry et al. [31]), ignores all inter-gene correlation except the correlation among the members of the tested gene set and estimates a single average pair-wise correlation for gene set members using residuals from a linear regression.

The PCGSE method makes similar simplifying assumptions as those made by CAMERA, i.e., correlation between the *z*_*j*_ can be approximated by correlation among the genomic variables, only gene set members have non-zero inter-gene correlation and all pair-wise correlations between gene set members are the same. An important difference between PCGSE and CAMERA is that PCGSE estimates the average inter-gene correlation directly from the sample correlation matrix. The correlation-adjusted standardized mean difference statistic used by PCGSE is:

(4) $$  S^{D,adj}_{k} = \frac{\bar{z}_{k} - \bar{z}_{k^{c}}}{\sigma_{p} \sqrt{\frac{\text{VIF}}{m_{k}} - \frac{1}{p-m_{k}}}}  $$

where VIF (variance inflation factor) $ = 1 + (m_{k} - 1) \bar {\rho }_{k}$ and $\bar {\rho }_{k}$ is the average Pearson correlation coefficient between members of gene set *k*. Following Wu et al. [32], this correlation-adjusted statistic has a t-distribution with *n*−2 df under *H*_0_. Likewise the correlation-adjusted standardized rank sum statistic is computed as:

(5) $$  S^{W,adj}_{k} = \frac{W_{k} - \mu_{W_{k}}}{\sigma^{2}_{\text{VIF},W_{k}}}  $$

where $\sigma ^{2}_{\text {VIF},W_{k}} = (m_{k}(p-m_{k}))/(2\pi) (sin^{-1} (1) + (p-m_{k} - 1) sin^{-1} (.5) + (m_{k}-1) (p-m_{k}-1)sin^{-1}(\bar {\rho }_{k}/2) + (m_{k}-1)sin^{-1}((\bar {\rho }_{k}+1)/2))$, as derived in Wu et al. [32] based on the formula in Barry et al. [31].

#### Permutation test

The most common approach in the gene set testing literature for addressing correlation between the *z*_*j*_ has been sample permutation. This approach, which corresponds to the class 2 test in Barry et al. [31], generates the null distribution of the *S*_*k*_ via permutation of the outcome variable. For each permutation of the outcome variable, all *z*_*j*_ are recomputed to generate permutation statistics $z^{*}_{j}$ and then permutation gene set statistics $S^{*}_{k}$ are calculated using the $z^{*}_{j}$. The statistical significance for a given gene set *k* is based on the proportion of all permutation $S^{*}_{k}$ more extreme than the observed *S*_*k*_. In standard gene set testing, permutation is applied to a clinical outcome variable, e.g., a case/control label.

For PCGSE, permutation is applied to the elements of the target PC, i.e., the elements of one of the columns of **U*Σ***. Because permutation is applied to the PC elements, this test can only be used with Pearson correlation coefficients or Fisher-transformed Pearson correlation coefficients as gene-level statistics since only these gene-level statistics can be recomputed after permutation of the PC elements (the PC loadings are fixed). A key assumption of the permutation null distribution is that the permuted values are i.i.d. Assuming the original *n* observations of the p-dimensional random vector **x** are i.i.d, the elements of each PC will also be i.i.d., since each PC is a linear function of the original **x**. Permutation of the PC elements therefore generates a valid permutation distribution for both ${S^{D}_{k}}$ and ${S^{W}_{k}}$.

Because permutation tests handle correlation among the *z*_*j*_ without attempting to estimate this correlation or make simplifying assumptions about the correlation structure, they are likely the most accurate of the statistical tests supported by PCGSE and are therefore used to evaluate the performance of the parametric and correlation-adjusted parametric tests. The exact permutation test was also used as a “gold-standard” in Zhou et al. [34]. Although they provide superior handling of inter-gene correlation, permutation tests do suffer from two important disadvantages relative to parametric tests: computational complexity and lower power to detect gene sets whose members all have a small common association with the outcome. Because of these disadvantages, correlation-adjusted parametric tests are preferred for most PCGSE applications.

Another alternative to sample permutation testing that addresses the key challenge of computational complexity is the parametric approximation of the sample permutation distribution of gene-level score statistics developed by Zhou et al. [34]. Although the Zhou et al. beta distribution-based parametric approximations may be a useful option for the PCGSE method, it is not currently supported due to the lack of a parametric approximation for a directional, competitive gene set test statistic that is equivalent to ${S^{D}_{k}}$ or ${S^{W}_{k}}$ using untransformed *z*_*j*_. In Zhou et al. [34], parametric approximations are only detailed for two self-contained gene set test statistics (sum of the score statistics and sum of the squares of the score statistics) and one non-directional competitive test statistic (a weighted sum of the squares of local score statistics).

### PCGSE evaluation

#### Benchmark PC gene set testing method

Because contingency table-based tests represent the current state-of-the-art for competitive gene set testing relative to the sample PCs of genomic data [30], it is important to compare the performance of the PCSGSE method, specifically the tests based on the *S*^*D*,*adj*^ and *S*^*W*,*adj*^ gene set statistics, against tests based on a 2×2 contingency table populated via a discretization of the ranked list of PC loading values. Since tests based on a discretization of the gene-level test statistics, e.g., Pearson’s difference in proportions test [31], are simply a special case of the unadjusted mean difference statistic, *S*^*D*^, the PCGSE method using the unadjusted t-test can be used as a proxy for existing contingency table methods in both the simulation and real data examples. Furthermore, because a two-sample t-test based on *S*^*D*^ is more powerful than the corresponding contingency table test based on a discretization of the gene-wise test statistics, this comparison is conservative, i.e., the difference in performance between the PCGSE method using *S*^*D*,*adj*^ or *S*^*W*,*adj*^ and contingency table tests should be greater than the difference between PCGSE using *S*^*D*,*adj*^ or *S*^*W*,*adj*^ and PCGSE using *S*^*D*^.

#### Evaluation using simulated gene sets and simulated data

**Fig. 2:**
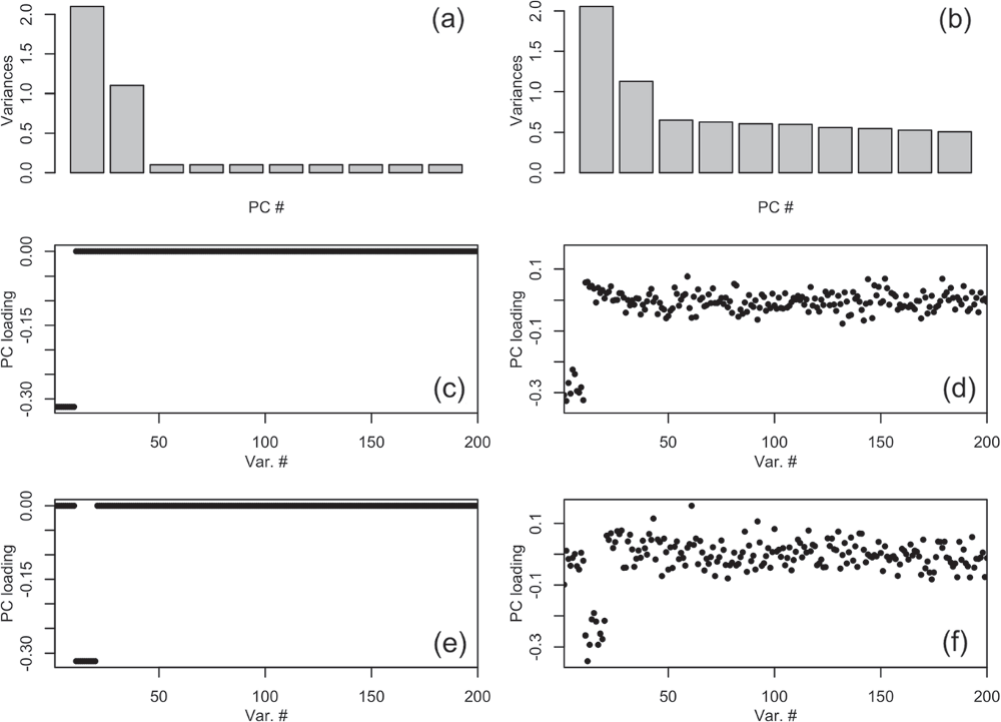
Simulation model. Variances and loadings for the principal components a 200-dimensional population covariance matrix, ***Σ***, and the sample covariance matrix estimated from n = 75 independent observations of the random vector **x**∼*MV* *N*(**0**,***Σ***) where ***Σ*** is generated according to the model outlined in Section “Benchmark PC gene set testing method”. Variances for the first ten population PCs are shown in plot (**a**) and loadings for the first two population PCs are shown in plots (**c**) and (**e**). Plots **b**, **d** and **f** show the corresponding variances and loadings for the sample PCs of a single simulated dataset

As a simple example, the PCGSE method was used to compute the statistical association between 20 disjoint gene sets, each of size 10, against the PCs of 1,000 simulated gene expression datasets each comprised by 75 independent observations of a 200-dimensional random vector simulated according to a multivariate normal distribution ∼MVN(***μ***,***Σ***). The population covariance matrix was generated as: $\boldsymbol {\Sigma } = \lambda _{1} \boldsymbol {\alpha }_{1} \boldsymbol {\alpha }_{1}^{T} + \lambda _{2} \boldsymbol {\alpha }_{2} \boldsymbol {\alpha }_{2}^{T} + \lambda _{d} \boldsymbol {I}$, where *λ*_1_= 2, *λ*_2_= 1, *λ*_*d*_= 0.1, ***α***_1_ is a 200-dimensional vector with all elements equal to 0 except for the first 10 which were set to $\sqrt {.1}$, ***α***_2_ is a 200-dimensional vector with all elements equal to 0 except for the second 10 which were set to $\sqrt {.1}$. Figure 2 graphically illustrates the variance and loadings of the population and sample PCs simulated according to this model. The PCGSE method was executed using the Fisher-transformed Pearson correlation coefficient between each variable and each PC as the *z*_*j*_ with ${S^{D}_{k}}$, as defined in (2), as the gene set test statistic. The statistical significance of the association between each of the 20 simulated gene sets and each PC was computed using all supported tests described in Section “Gene set statistical significance” with 10,000 permutations for the permutation tests. Because the true association was known between simulated gene sets and the PCs of the simulated data, it was possible to compute contingency table statistics. In this case, the type I error rates for the different statistical testing methods were computed for gene set 2 relative to PC 1 and for gene set 1 relative to PC 2, both cases with no true association.

#### Evaluation using Spellman et al. *α* factor-synchronized yeast gene expression data and yeast cell cycle gene sets

The PCGSE method was used to compute the statistical association of the yeast cell cycle gene sets defined by Spellman et al. [39] relative to the first three PCs of a specially processed version of the *α* factor-synchronized yeast gene expression data collected by Spellman et al. and re-examined by Alter et al. [5]. Both the *α* factor-synchronized data and yeast cell cycle gene sets were downloaded from the Additional file 1 website for Alter et al. To support comparison against the results reported in Alter et al., PCA was performed on a version of the gene expression data that was specially processed according to the steps outlined in Alter et al. so that the first three PCs were identical to the first three so-called eigengenes. A reproduction of Fig. 5 from Alter et al. is included as Fig. 2 in Additional file 1 with the value of the first three PCs of the specially processed gene 350 expression data shown relative to the 22 *α* factor arrays. The PCGSE method was executed on the Spellman et al. data and gene sets using the Fisher-transformed Pearson correlation coefficient between each gene and each PC as the *z*_*j*_ with ${S^{D}_{k}}$ as the gene set statistic. The statistical significance of the gene set statistic was computed using all supported tests described in Section “Gene set statistical significance” with 10,000 permutations for the permutation tests.

#### Evaluation using MSigDB C2 v4.0 gene sets and Armstrong et al. leukemia gene expression data

The PCGSE method was also used to compute the statistical association between the MSigDB C2 v4.0 gene sets and the first 3 PCs of the leukemia gene expression data [40] used in the 2005 GSEA paper [35]. The MSigDB C2 v4.0 cancer modules and collapsed leukemia gene expression data were both downloaded from the MSigDB repository. With a minimum gene set size of 15 and maximum gene set size of 200, 3,076 gene sets out of the original 4,722 were used in the analysis. The PCGSE method was executed using the Fisher-transformed Pearson correlation coefficient between each genomic variable and each PC as the *z*_*j*_ and ${S^{D}_{k}}$ as the gene set test statistic. The statistical significance of the association between each of the MSigDB C2 gene sets and each of the first 3 PCs of the standardized leukemia gene expression data was computed using all supported tests described in Section “Gene set statistical significance” with 10,000 permutations for the permutation tests. The enrichment of the MSigDB C2 gene sets was also computed relative to the acute myeloid leukemia (AML) versus acute lymphoblastic leukemia (ALL) phenotype using the GSA method [36] with the restandardized mean statistic and 10,000 permutations. For each of the first three PCs and each of the PCGSE methods for computing statistical significance of the standardized mean difference gene set statistic, the Spearman correlation coefficient was computed between PC gene set enrichment p-values and phenotype enrichment p-values. For PC 2, for which the PC and phenotype gene set enrichment p-values were highly correlated, contingency table statistics were computed measuring how well PCGSE was able to identify MSigDB C2 gene sets significantly associated with the AML/ALL phenotype.

## Results and discussion

### Simulation example

According to the population covariance matrix, ***Σ***, used to simulate the 1,000 datasets, only the first gene set should be significantly enriched on the first PC and only the second gene set should be significantly enriched on the second PC. This relationship can be seen easily in the loading values for population PCs 1 and 2 as shown in Fig. 2, plots **(****c****)** and **(****e****)**. The significant loading of gene set 2 on PC 2, however, will result in a high pair-wise correlation between the PC loadings for gene set 2 members on PC 1. The fact that high loadings on one PC result in correlation among the PC loadings on other PCs follows from the formula for the asymptotic distribution of the PC loadings for MVN data [38]: $\mathbf {v}_{j} \sim \mathcal {N}(\boldsymbol {\alpha }_{j}, \mathbf {T}_{j}), \mathbf {T}_{j} = \frac {\lambda _{j}}{n-1} \sum _{k=1, k \neq j}^{p} \frac {\lambda _{k} \boldsymbol {\alpha _{k} \alpha _{k}}^{T}}{(\lambda _{k}-\lambda _{j})}$, where *j*=1,…,*p*, *p* is fixed and *n*→*∞*, *λ*_*j*_ is an eigenvalue of the population covariance matrix, *λ*_1_>*λ*_2_>…>*λ*_*p*_, and ***α***_*j*_ is an eigenvector of the population covariance matrix.

**Fig. 3:**
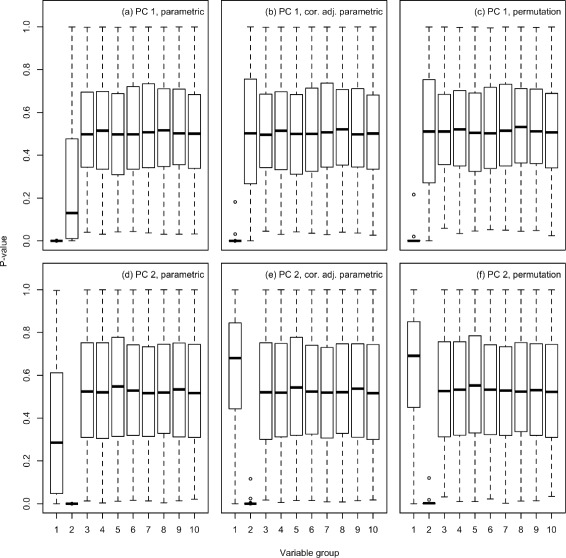
Simulation results for standardized mean difference statistic, ${S_{k}^{D}}$. Boxplots showing the distribution of PCGSE-computed enrichment p-values for the first 10 of 20 simulated gene sets relative to the first 2 PCs of 1000 datasets simulated according to the model described in Section “Benchmark PC gene set testing method” of the main PCGSE manuscript and illustrated in Fig. 2 above. For all displayed results, PCGSE was executed using the Fisher-transformed Pearson correlation coefficient between each genomic variable and each PC as the gene-level test statistic with the standardized mean difference as the gene set test statistic. Plots **a**, **b** and **c** display the distribution of enrichment p-values for the first 10 gene sets relative to the first PC of all simulated data sets. In plots **d**, **e** and **f**, enrichment p-values computed relative to the second PC are displayed. For plots **a** and **d**, the p-values were computed using a two-sided t-test on ${S^{D}_{k}}$, for plots **b** and **e**, the p-values were computed using a two-sided t-test on $S^{D,adj}_{k}$ and, for plots **c** and **f**, the p-values were computed using a two-sided permutation test on ${S^{D}_{k}}$. For PC 1 and gene set 2, the type I error rate at a nominal *α* of 0.05 was 0.382 for the unadjusted t-test, 0.057 for the correlation-adjusted t-test and 0.05 for sample permutation of ${S_{k}^{D}}$. For PC 2 and gene set 1, the type I error rate at a nominal *α* of 0.05 was 0.257 for the t-test, 0.016 for the correlation-adjusted t-test and 0.014 for sample permutation test

The gene-level test statistics computed for gene set 2 on PC 1 and for gene set 1 on PC 2 will therefore have a non-zero average pair-wise correlation. The impact of this correlation between the gene-level test statistics can be seen in the PCGSE results shown in Fig. 3. The unadjusted t-test uses an incorrectly small variance for the ${S^{D}_{k}}$ gene set statistic and, as expected, generates the high type I error rate of 0.382 given a nominal *α* of 0.05 for gene set 2 relative to PC 1 and 0.257 for gene set 1 relative to PC 2. The correlation-adjusted two-sided t-test and the two-sided permutation test are much more successful at controlling the type I error rate. For PC 1 and gene set 2, the type I error rate was 0.057 for the correlation-adjusted t-test and 0.05 for the permutation test. For PC 2 and gene set 1, the type I error rate was 0.016 for the correlation-adjusted t-test and 0.014 for the permutation test. For this example, all gene set testing methods were able to correctly reject the null hypothesis for almost all cases where the gene set had a true association with the PC, e.g., gene set 1 relative to PC 1 and gene set 2 relative to PC 2. When this simulation is performed with smaller sample sizes, both the type I and type II error rates increase due to increased uncertainty in the estimation of the sample covariance matrix and sample PCs (results not shown). The lower type I error rates for gene set 1 relative to PC 2 versus gene set 2 relative to PC 1 can be explained by the larger uncertainty in the estimation of the eigenvector for the second PC relative to the first PC and the consequent overestimation of correlation between gene-level test statistics. PCGSE results computed for this simulation example using ${S^{W}_{k}}$ as the gene set statistic can be found in the Additional file 1.

Although based on a simple two-factor MVN model, this simulation example demonstrates the importance of controlling for correlation between gene-level test statistics. Tests which assume independence among the statistics that quantify the association between genes and PCs, such as a two-sample t-test, Fisher’s exact test or a gene permutation test, will underestimate the variance of the gene set test statistic and will therefore reject too many *H*_0_. This example also shows that the correlation-adjusted t-test can achieve enrichment sensitivity and specificity comparable to a sample permutation test with a lower computational burden.

### Yeast cell cycle gene expression example

The Spellman et al. [39] *α* factor-synchronized gene expression data was selected for PCGSE analysis because it is easily accessible, has been widely reanalyzed and has a spectra with a published biological interpretation. In particular, the reanalysis by Alter et al. [5] was one of the first to illustrate that the spectra of gene expression data can represent important biological features, in this case phases of the yeast cell cycle. In Alter et al., the authors provided a qualitative interpretation of the first two eigengenes in terms of the yeast cell cycle by examining the correlation between the eigengenes and genes known to be active during different cell cycle phases, as defined by Spellman et al. Alter et al. concluded that the first eigengene was correlated with genes that peak late in cell cycle phase *G*_1_ and early in phase *S* and was anticorrelated with genes that peak late in cell cycle phase *G*_2_/*M* and early in phase *M*/*G*_1_. Alter et al. also concluded that the second eigengene was correlated with genes that peak late in cell cycle phase *M*/*G*_1_ and early in phase *G*_1_ and was anticorrelated with genes that peak late in phase *S* and early in phase *S*/*G*_2_.

Table 1 contains p-values representing the statistical significance of the association between each of the Spellman et al. [39] yeast cell cycle gene sets and the first two PCs of a specially processed version of the Spellman et al. gene expression data. As described in Section “Evaluation using Spellman et al. *α* factor-synchronized yeast gene expression data and yeast cell cycle gene sets”, this special processing ensured that the PCs were identical to the eigengenes analyzed in Alter et al. [5]. When a two-sided t-test was used to determine the statistical significance of the ${S^{D}_{k}}$ gene set statistic, the gene sets corresponding to cell cycles *G*_1_, *S* and *G*_2_/*M* were highly significantly associated with PC 1 and the gene sets corresponding to M/ *G*_1_, *G*_1_, *S* and *S*/*G*_2_ were significantly associated with PC 2. However, when either a two-sided t-test was used to compute the significance of $S^{D,adj}_{k}$ or a two-sided permutation test was used to determine the statistical significance of ${S^{D}_{k}}$, PC 1 only had a significant association with the gene set corresponding to phase *G*_1_ (with a marginally significant association with phase *G*_2_/*M*) and none of the cell cycle gene sets were significant for PC 2.

**Table 1:** Yeast cell cycle results. PCGSE computed enrichment p-values for the Spellman et al. [39] yeast cell cycle gene sets relative to the first two PCs of the Spellman et al. *α* factor-synchronized gene expression data processed using the steps outlined in Alter et al. [5]. PCGSE was executed using Fisher transformed Pearson correlation coefficients between genes and PCs as gene-level test statistics

	T-test		Cor-adj t-test		Perm	
	PC 1	PC 2	PC 1	PC 2	PC 1	PC 2
*M*/*G*_1_	0.68	**1.2e-12**	0.94	0.18	0.94	0.22
*G*_1_	**3.5e-130**	**1e-35**	**0.023**	0.23	**0.024**	0.35
*S*	**1e-10**	**0.0074**	0.2	0.59	0.2	0.62
*S*/*G*_2_	0.27	**4.6e-06**	0.86	0.45	0.87	0.47
*G*_2_/*M*	**8.3e-38**	0.068	0.07	0.79	**0.048**	0.81

Significance of the ${S^{D}_{k}}$ gene set statistic was computed using either a two-sided t-test, a correlation-adjusted two-sided t-test or a two-sided permutation test

Unadjusted p-values less than 0.05 are displayed in bold

Comparing the output from PCGSE with the analysis in Alter et al. [5], the results from the two-sided t-test align closely with the qualitative conclusions of Alter et al. The output from the correlation-adjusted t-test and permutation test, although generally in agreement for PC 1, are in direct contrast with Alter et al. regarding PC 2, finding no cell cycle association. The agreement between Alter et al. and the t-test results is expected since the authors had based their analysis simply on a qualitative inspection of the gene-level correlations without a more formal test of a gene set test statistic. The fact that the PCGSE methods which account for inter-gene correlation failed to find an association between PC 2 and the cell cycle gene sets indicates that the published association in Alter et al. may well have been a false positive due to either the high inter-gene correlation present among the members of these sets or the selective examination by Alter et al. on a subset of the genes in each of the cell cycle gene set with a common direction of association with the eigengene. In the later case, it is likely that a gene set statistic such as the maxmean [36] would identify significant cell cycle enrichment for the second eigengene.

This example highlights the importance of using formal statistical methods for gene set testing when attempting to interpret the PCs of genomic data in terms of gene sets. Such gene set testing methods must specifically account for the correlation between gene-level test statistics.

### Leukemia gene expression example

The classic Armstrong et al. [40] leukemia gene expression dataset is another excellent example of a case where the genomic patterns associated with an interesting phenotype have a clear representation in the spectral structure of the data. For the Armstrong et al. data, the second PC of the gene expression data is strongly associated with the AML versus ALL status of the subjects. Use of the Armstrong et al. gene expression data and MSigDB C2 v4.0 gene sets for evaluation of PCGSE was also motivated by the extensive use of this dataset and gene set collection in the gene set enrichment literature (e.g., Subramanian et al. [35]) and easy accessibility from the MSigDB repository, factors that will facilitate interpretation and replication of the reported PCGSE results by other researchers.

**Fig. 4:**
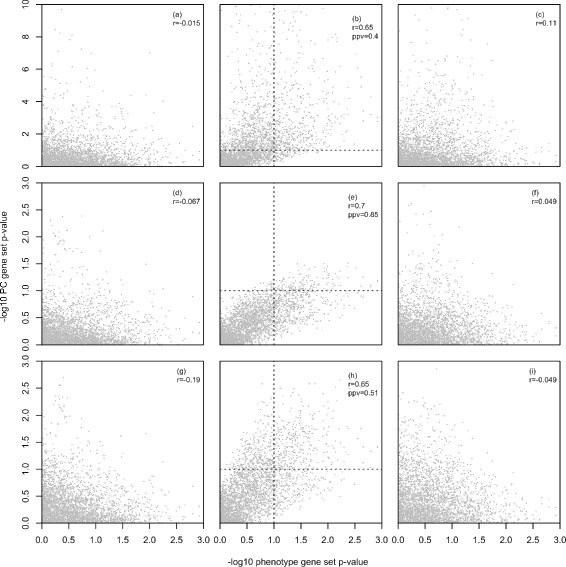
Leukemia gene expression results. Scatter plots showing the association between phenotype gene set enrichment p-values and PC gene set enrichment p-values for the Armstrong et al. [40] leukemia gene expression data, AML/ALL phenotype, MSigDB C2 v4.0 gene sets and first three PCs. Both phenotype and PC gene set enrichment p-values were computed as outlined in Section “Evaluation using MSigDB C2 v4.0 gene sets and Armstrong et al. leukemia gene expression data”. Shown in each plot is the Spearman correlation coefficient between phenotype and PC gene set enrichment p-values and the positive predictive value of PC gene set enrichment for identifying gene sets that are significantly enriched relative to the phenotype at an *α*=0.1 (shown by *dotted lines*). True positives are in the upper right quadrant, false positives are in the upper left quadrant. Plots **a**-**c** show the association between phenotype and PC gene set enrichment p-values for PCs 1 through 3 with the PC enrichment p-values computed using a two-sided t-test on the standardized mean difference gene set statistic. For plots **d**-**f**, the PC gene set enrichment p-values were computed using a correlation-adjusted two-sided t-test and, for plots **g**-**i**, the PC gene set enrichment p-values were computed using the permutation distribution of the gene set statistic

Figure 4 shows the association between phenotype and PC gene set enrichment p-values for the MSigDB C2 v4.0 gene sets, the AML versus ALL phenotype and the first three PCs of the Armstrong et al. leukemia gene expression data. Each of the columns in the multi-plot corresponds to results for one PC and each row corresponds to one of the three different statistical tests supported by PCGSE on the ${S^{D}_{k}}$ gene set statistic (i.e., t-test, correlation-adjusted t-test and permutation test). The association between PC 2 and the AML versus ALL phenotype can be clearly seen in Fig. 4 plots (b), (e) and (h). For all three PCGSE methods, the PC enrichment p-values for the MSigDB C2 v4.0 gene sets are highly correlated with the enrichment p-values computed for these gene sets relative to the AML versus ALL phenotype.

Similar to the PCGSE results outlined in previous sections on simulated data and yeast gene expression data, the unadjusted two-sided t-test on ${S^{D}_{k}}$ generates PC gene set enrichment p-values that are substantially lower than the enrichment p-values output by either the t-test on $S^{D,adj}_{k}$ or the permutation test on ${S^{D}_{k}}$. Although the true enrichment status of the MSigDB C2 v4.0 gene sets relative to the PCs of the Armstrong et al. [40] gene expression data is unknown, the phenotype enrichment results can be used as a proxy for the true gene set association with PC 2 under the assumption that this PC captures the AML versus ALL signal. If gene sets with a phenotype enrichment significance at or below 0.1 are considered AML/ALL markers, the PCGSE method is able to correctly identify these gene sets via enrichment relative to PC 2 with an area under the receiver operator characteristic curve (AUC) of 0.82 for the t-test results displayed in plot (b), an AUC of 0.88 for the correlation-adjusted t-test results displayed in plot (e) and an AUC of 0.83 for the permutation test results displayed in plot (h). Considering identification of AML/ALL-associated gene sets via PC enrichment using just *α*= 0.1, the PCGSE method has a positive predictive value of 0.4 for the t-test results displayed in plot (b), 0.85 for the correlation-adjusted t-test results displayed in plot (e) and 0.51 for the permutation test results displayed in plot (h).

PCGSE analysis of the MSigDB C2 v4.0 gene sets and Armstrong et al. leukemia gene expression data illustrates the biological motivation for PC gene set enrichment and demonstrates the superior performance of the computationally efficient correlation-adjusted t-test relative to either an unadjusted t-test or permutation test.

## Conclusion

Although PCA is widely used for the dimensional reduction of biomedical data, with applications in visualization, clustering and regression, interpretation of PCA-based models remains challenging. While rotation methods and sparse PCA techniques can generate approximate PCs with few non-zero loadings that support interpretation in terms of individual variables, these approaches will perform poorly on genomic data in which important biological signals are defined by the collective action of groups of functionally related genes. Although gene set testing methods have been widely applied in supervised settings to analyze the association between gene sets and clinical phenotypes, such variable group testing methods have seen little application in unsupervised contexts to test the association between gene sets and the spectra of genomic data. To address the challenge of gene set-based interpretation of the PCs of genomic data, we have developed the principal component gene set enrichment (PCGSE) method, available as an R package from CRAN. PCGSE performs a two-stage competitive gene set test with the correlation between each gene and each PC as the gene-level test statistic and with the flexible choice of both the gene set test statistic and the method used to compute the null distribution of the gene set statistic. On both simulated gene sets with simulated data and on curated gene sets with real gene expression data, a computationally efficient version of the PCGSE method based on a correlation-adjusted t-test has been shown to accurately compute the statistical association between gene sets and the PCs of genomic data. Methods for combining the results from PCGSE tests on multiple PCs will be explored in future work.

## Availability of supporting data

The MSigDB C2 v4.0 gene sets can be downloaded from http://www.broadinstitute.org/gsea/msigdb/collections.jsp. The Armstrong et al. [40] leukemia gene expression data can be downloaded from http://www.broadinstitute.org/gsea/datasets.jsp. The Spellman et al. [39] yeast cell cycle data can be downloaded from http://genome-www.stanford.edu/SVD/htmls/pnas.html. An implementation of the PCGSE algorithm is available in the PCGSE R package (http://cran.r-project.org/web/packages/PCGSE/index.html). Due to the dependency on the Bioconductor package safe, it is recommended that PCGSE be installed using the biocLite() function. At the R prompt, enter: source(“http://bioconductor.org/biocLite.R”) biocLite(“PCGSE”)

## Additional file

Additional file 1 **Supplementary figures for simulation and cell cycle examples.** (PDF 698 kb)
