# Supplementary material for: Principal component gene set enrichment (PCGSE)
Source: BioData Min. 2015 Aug 19;8:25. doi: 10.1186/s13040-015-0059-z (PMC4543476; doi:10.1186/s13040-015-0059-z)
Supplement: Additional file 1 — Supplementary figures for simulation and cell cycle examples. (PDF 698 kb) [file 13040_2015_59_MOESM1_ESM.xls]

Principal component gene set enrichment (PCGSE) Supplementary Material

H. Robert Frost, Zhigang Li and Jason H. Moore

1 Supplemental results for simulation example

Figure 1 below displays the PCGSE results for the simulation example discussed in Sections 2.8.1 and 3.1 of the main manuscript when using S_k^W as the gene set statistic.

Note that the results for S_k^W using parametric and permutation tests are similar to those for S_k^D. Although the correlation-adjusted z-test based on the $S_k^{W,adj}$ has an improved type I error rate relative to the unadjusted z-test, it has an inflated type II error rate. The inflated type II error rate for the correlation-adjusted z-test is likely due to an overestimated VIF under the alternative hypothesis, as computed by the equation from Barry et al. [1]. Therefore, in cases where a rank sum gene set statistic is motivated, PCGSE should be performed using a permutation test and not using the more efficient correlation-adjusted z-test.

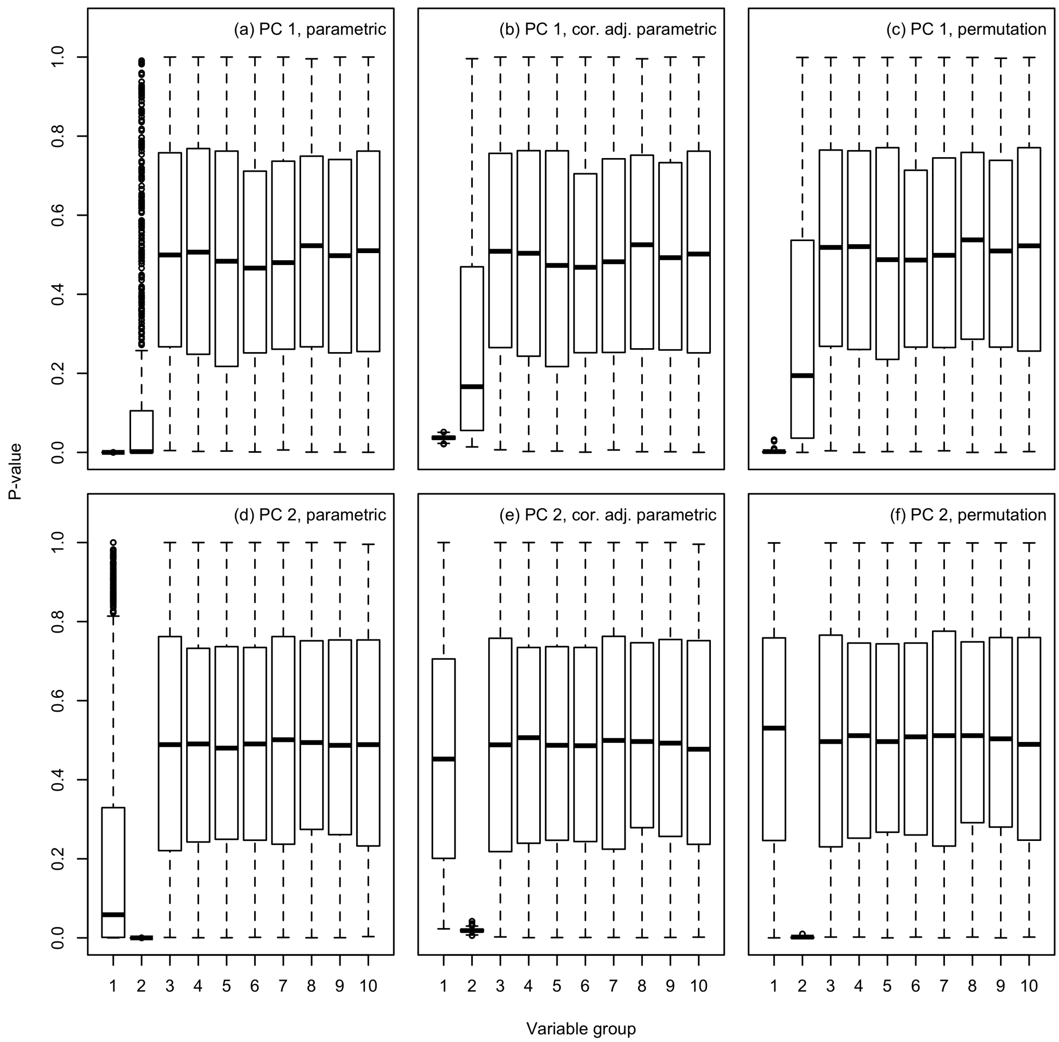

Figure 1: Simulation results for standardized rank sum statistic, S_k^W. Distribution of PCGSE-computed enrichment p-values for the first 10 of 20 simulated gene sets relative to the first 2 PCs of 100 datasets simulated according to the model described in Section 2.8.1 of the main PCGSE manuscript and illustrated in supplemental Figure 1 above. For all displayed results, PCGSE was executed using the Fisher-transformed Pearson correlation coefficient between each genomic variable and each PC as the gene-level test statistic and the standardized rank sum statistic as the gene set test statistic. Plots **(a)**, **(b)** and **(c)** display the distribution of enrichment p-values for the first 10 gene sets relative to the first PC of all simulated data sets. In plots **(d)**, **(e)** and **(f)**, enrichment p-values computed relative to the second PC are displayed. For plots **(a)** and **(d)**, the p-values were computed using a two-sided z-test on S_k^W. for plots **(b)** and **(e)**, the p-values were computed using a two-sided z-test on $S_k^{W,adj}$ and, for plots **(c)** and **(f)**, the p-values were computed using a two-sided permutation test on $S_k^{W,adj}$. For PC 1 and gene set 2, the type I error rate at a nominal α of 0.05 was 0.687 for the unadjusted z-test, 0.223 for the correlation-adjusted z-test and 0.282 for sample permutation of the rank sum statistic. For PC 2 and gene set 1, the type I error rate at a nominal α of 0.05 was 0.485 for the z-test, 0.028 for the correlation-adjusted z-test and 0.07 for sample permutation of the rank sum statistic.

2 Supplemental results for yeast cell cycle example

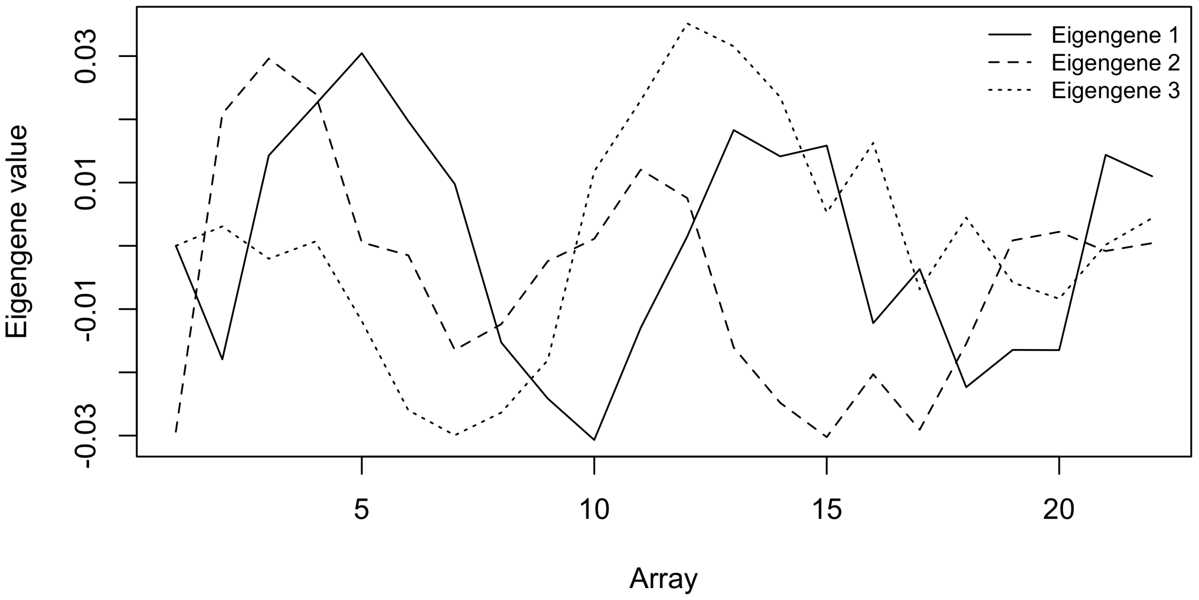

Figure 2: Reproduction of Figure 5 from Alter et al. [2] that displays the value of first three PCs of the specially processed gene expression matrix, i.e., the "eigengenes", over the 22 α factor-synchronized arrays. According to Alter et al., the following approximate mapping held between arrays and cell cycle phases: 1 (NA), 2 (M/G1), 3,4 (G1), 5,6 (S), 7,8 (S/G2), 9 (G2/M), 10,11 (M/G1), 12,13 (G1), 14,15 (S), 16 (S/G2), 17,18 (G2/M), 19,20 (S/G1), 21,22 (G1)

References

[1] Barry, W.T., Nobel, A.B., Wright, F.A.: A statistical framework for testing functional categories in microarray data. Ann Appl Stat **2**, 286–315 (2008)

[2] Alter, O., Brown, P.O., Botstein, D.: Singular value decomposition for genome-wide expression data processing and modeling. Proc Natl Acad Sci USA **97**(18), 10101–10106 (2000)
